# Effects of ^13^C isotope-labeled allelochemicals on the growth of the invasive plant *Alternanthera* *philoxeroides*

**DOI:** 10.1038/s41598-023-39889-7

**Published:** 2023-08-23

**Authors:** Zexun Hua, Qingsong Xie, Yue Li, Mengying He, Yan Wang, Hongmiao Wu, Zhen Zhang

**Affiliations:** College of Resources and Environment, Anhui Agricultural University, Hefei, 230036 China

**Keywords:** Ecology, Plant sciences

## Abstract

The secondary metabolites of indigenous plants have significant allelopathic inhibitory effects on the growth and development of invasive alien plants. Methyl palmitate (MP) and methyl linolenate (ML) were used as exogenous allelopathic substances. The research investigated the differences of inhibitory effects of MP and ML on the growth of seedlings of *Alternanthera* *philoxeroides*, and calculated their morphological characteristics, biomass, physiological indicators and the response index (RI). The synthetical allelopathic index (SE) of 1 mmol/L MP was the smallest (− 0.26) and the allelopathic inhibition was the strongest; therefore, it was selected as a ^13^C-labeled allelochemical. The distribution of 1 mmol/L MP in different parts of *A.* *philoxeroides* and the correlation between the biomass ratios of roots, stems and leaves and the ^13^C content were studied by ^13^C stable isotope tracing experiments. Atom percent excess (APE) between roots, stems and leaves of *A.* *philoxeroides* treated with 1 mmol/L MP were significantly different in terms of magnitude, with leaves (0.17%) > roots (0.12%) > stems (0.07%). The root, stem and leaf biomass ratios of invasive weeds had great significant positive correlation with ^13^C content (*p* < 0.01, R^2^ between 0.96 and 0.99). This current research provides a new idea and method for the control of *A.* *philoxeroides*, but large-scale popularization remains to be studied.

## Introduction

In the background of global climate change, invasive exotic plants are one of the main drivers of biodiversity loss and pose a serious threat to ecosystem stability^1^. Invasive plants successfully invaded the invasive sites through a variety of competitive ways, showing higher phenotypic plasticity^2^. Invasive plants have high energy utilization efficiency and low reproductive cost, which make them grow and develop rapidly and have stronger chemical defense ability^3^. The "new weapon hypothesis" suggests that invasive plants produce allelochemicals in various ways, such as root secretions, to inhibit the growth and development of other plants, thus achieving invasion^4,5^.

*Alternanthera* *philoxeroides*, a perennial herb of the genus Amaranthaceae, is on the first list of invasive alien species in China and is highly adaptable and phenotypically plastic^6^. Nevertheless, their asexual reproduction and competitive ability can lead to the loss of local species diversity, homogenize species, and cause crop yield reduction^7^. The effectiveness of the physical, chemical and biological control of *A.* *philoxeroides* has been too costly in human and environmental terms^8^. Native plants can well control the growth of *A.* *philoxeroides* through replacement control, and aqueous extracts of *Humulus* *scandens* roots, *Ipomoea* *batatas*, and *Phragmites* *australis* organs reduce invasive spread by inhibiting their photosynthesis and seedling development, resulting in effective management^9–11^.

Indigenous plants can inhibit and repel the growth and expansion of invasive plants by secreting specific metabolites into the surrounding environment to produce allelopathy, which can lower plant performance by roughly 25%^12,13^. Allelochemicals are the main material of allelopathy^14^. Most of the allelochemicals produced by plants are secondary metabolites, which are classified into fourteen categories according to their structural function and composition, the most common of which includes organic acids, phenols and terpenoids^15^. Allelopathic substances are largely released to the surrounding environment through rain and fog leaching, natural volatilization, plant remnants and litter decomposition, and root secretion, while plants normally release secondary metabolites in one or more ways^16^. Physiological activities such as cell division, membrane permeability, photosynthesis and respiration, enzyme synthesis and metabolism, proteins and nucleic acids in plants are all closely related to allelopathic substances^17^. For example, peach root bark extract and benzoic acid can diminish photosynthetic parameters and increased antioxidant enzymes in peach seedlings, resulting in loss of organelles and nuclear irregularities^18^. Changes in cell permeability, cell damage, altered membrane permeability, reduced mineral uptake and embryo water absorption in bread wheat seedlings due to steroids and phenolic compounds in weed aqueous extracts^19^.

Fatty acid methyl esters have strong affinity for ester compounds and can be used as herbicides and herbicide carriers to remove weeds^20,21^. Methyl palmitate (MP) and methyl linolenate (ML) belong to fatty acid methyl esters, which are natural secondary metabolic compounds that can be extracted from plants. They are contained in the aqueous extracts of *Humulus* *scandens*, *Sorghum*, *Alfalfa*, *Cassiae* and other plants^9,22–24^. It has been studied that MP and ML from *Carica* *papaya*, *Lantana* *camara*, *Pinus* *roxburghii* and *Rhazya* *stricta* extracts could effectively inhibit the seed germination rate of field weeds such as *Avena* *fatua*, *Euphorbia* *helioscopia*, *Chenopodium* *album*, *Phalaris* *minor*, and *Rumex* *dentatus*, with allelopathic potential^25^. We can use the allelopathic inhibition of indigenous plants as an alternative control to effectively inhibit the growth of invasive weeds.

Studies have shown that the allelochemicals secreted by plants can change soil pH, soil microbial environment, affect soil organic matter, lead to the deterioration of the living environment of other populations, and indirectly affect the growth of plants^26^. It can also be directly absorbed by root cells of other plants, affecting plant enzyme activities, reducing respiration and photosynthesis, and increasing cell membrane permeability, resulting in impaired plant physiology^27,28^. ^13^C stable isotope tracer technology is an effective means to research the mode of action of allelopathic substances on plants (indirect or direct action) and the dynamic changes of uptake, transport, synthesis and distribution in plants and the strength of allelopathic inhibition in different parts of the plant, and further reveal the allelopathy mechanism of allelochemicals inhibiting the growth of invasive weeds^29–31^. Han et al. found that different times and different concentrations of CO_2_ treatments affected the rate of ^13^C uptake in the root zone of oriental melon seedlings and altered the assimilation and distribution of ^13^C in stems and leaves^32^.

Many studies have explored the effects of aqueous extracts from different parts of the plant on *A.* *philoxeroides*. At present, there are few reports on the mode of allelopathic inhibition and assimilation and distribution of exogenous allelochemicals in water extracts that inhibit the growth of *A.* *philoxeroides* in different parts of the plant. The experiments were based on the analysis of the composition of the aqueous extracts of *Humulus* *scandens* roots in our project, and the fatty acid methyl esters of the natural extracts of native plants with higher content and stronger allelopathic inhibition, namely methyl palmitate and methyl linolenate, were selected as the treatment groups^9,33,34^*.* The ^13^C isotope tracer technique was applied to label 1 mmol/L MP, which has a stronger inhibitory effect on *A.* *philoxeroides*. We intend to investigate the effect of exogenous allelopathic substances on *A.* *philoxeroides* and discover the most cost-effective method to regulate its growth. ​Current research focuses primarily on four specific issues: (1) do exogenous allelochemicals MP and ML have allelopathic inhibitory effects on the growth of *A.* *philoxeroides*? (2) How do the exogenous allelochemicals MP and ML affect the morphological characteristics, biomass changes, and physiological indicators of *A.* *philoxeroides*? (3) What is the allelopathic response of *A.* *philoxeroides* to exogenous allelopathic substances? (4) Dose the ^13^C-labeled MP affect *A.* *philoxeroides* in an indirect or direct manner and what is their distribution in different parts of the plant?

## Material and methods

### Source of experimental materials

The invasive plant, *Alternanthera* *philoxeroides*, was used for the experiment, and the plant specimen number is ANUB001553. We comply with relevant institutions, national and international standards and laws in the collection of plants. *A.* *philoxeroides* with good growth and maturity were dug up in Nongcui Garden of Anhui Agricultural University (31° 52′ N, 117° 16′ E) and washed in the plant physiology laboratory. *A.* *philoxeroides* of uniform size and diameter of about 3 mm were selected, cut into rhizomes of length 3 cm along the stem nodes with scissors, disinfected in 10% solution of sodium hypochlorite for 10 min, and rinsed in distilled water to prevent mildew during their cultivation. Various circular sponges with a diameter of about 15 cm were selected, and taken holes evenly and place them in the bottom 1/5 of a 500 mL beaker to fix the *A.* *philoxeroides* and conserve water. The reagents methyl palmitate (MP) with purity > 99% and methyl linolenate (ML) with purity > 99.5% were purchased from Shanghai Titan Scientific Co., Ltd and Hefei Shang Cheng experiment supplies Ltd.

### Preparation of exogenous allelochemicals solutions

The solid powders of MP and ML were weighed on a ten thousand scale and emulsified in a beaker with distilled water in a constant temperature water bath at 40 °C with stirring. After cooling, it was shaken in the ultrasonic cleaner for 5 min to form an emulsion^35^. The emulsion was fixed in a 1000 mL volumetric flask, transferred to a wide-mouth flask and configured as 0.01, 0.1, 0.5, 1 mmol/L of MP (abbreviated as MP0.01, MP0.1, MP0.5, MP1) and ML (abbreviated as ML0.01, ML0.1, ML0.5, ML1) of which ^13^C-labeled MP1 solution (purchased from cambridge isotope laboratories inc, chemical purity ≥ 98%) solutions and stored in a refrigerator at 4 °C for backup. Distilled water was used as a control treatment.

### Hydroponic experiment

A double layer of filter paper was laid in the petri dishes and a small amount of distilled water was added. 10 rhizomes of *A.* *philoxeroides* were placed in each petri dish and incubated in a light incubator (SPX-250, Shanghai) (25 °C, 12 h D/12 h L, 80% relative humidity) for 7 days. The rhizomes of *A.* *philoxeroides* germinated and grew into seedlings. The round sponges were soaked in distilled water to absorb water and placed in 500 mL beakers. The experiment was conducted in a randomized complete block design (RCBD). 5 seedlings of *A.* *philoxeroides* were transplanted in each beaker at the perforation of the sponge, and the seedlings were cultivated in a light incubator for 45 days. Every 2 days, 5 mL of different concentrations of MP and ML solutions and control distilled water were provided to the seedlings, and the growth of *A.* *philoxeroides* was observed daily.

### Growth bioassay

At the end of the experiment, the morphological characteristics, biomass changes and physiological indicators of *A.* *philoxeroides* were measured. The roots, stems and leaves were cut and put into envelope bags respectively, and the root length and stem length were measured with a steel ruler, and the leaf area was measured with a leaf area meter (Yaxin-1241 leaf area meter, Beijing Yaxin LIYI Technology Co., LTD), and the number of nodes and leaves were counted. After the experiments were completed, the fresh weights of the shoot and root parts of the grasses were weighed and placed in an oven at 70 °C for 48 h to dry to a constant weight, and the dry weights of the shoot and root parts were weighed. Total chlorophyll, chlorophyll a, b and carotenoids contents were determined by colorimetric method using ethanol extraction^36^. The mid-vein of the leaves of *A.* *philoxeroides* was removed, 0.2 g of the fresh cut sample was weighed, a little CaCO_3_ and quartz sand were added, then 10 mL of anhydrous ethanol was added, and the plant tissue was ground until it turned white. After resting, the solution was filtered and set the volume to be 50 mL with anhydrous ethanol, a small amount of pigment extract was rinsed on the cuvette. The solution was added to 4/5 of the cuvette, and anhydrous ethanol was used as a blank control. The absorbance was determined colorimetrically using a UV spectrophotometer (T6 New Century, Beijing General Analytical Instrument Co.) tuned to the corresponding wavelength. Finally, the relevant chlorophyll content was calculated by the formula. We weighed 0.3 g of fresh leaves, crushed and centrifuged, placed in an ice–water mixture, and the supernatant was taken. The malondialdehyde (MDA) content, catalase (CAT), peroxidase (POD) and superoxide dismutase (SOD) activity indexes of *A.* *philoxeroides* leaves were determined by the kit (Nanjing Jiancheng)^37^. Related indicator formula:

1 $${\text{Root-shoot ratio}} = {\text{Root dry weight}}/{\text{ Shoot dry weight}}$$

2 $${\text{Plant water content}} = ({\text{fresh weights}} - {\text{dry weights}})/{\text{fresh weights}}$$

3 $${\text{Degree of succulence = fresh weights}}/{\text{ dry weights}}$$

Calculation formula for chlorophyll and carotenoids^36^:

4 $${\text{Chl. a}} = 13.95{\text{A}}_{663} - 6.8{\text{A}}_{645}\,({\text{mg}}/{\text{g}})$$

5 $${\text{Chl. b}} = 24.96{\text{A}_{645}} - {\text{7.32}}\text{A}_{663}({\text{mg}}/{\text{g}})$$

6 $${\text{Total Chl. Content}} = {\text{Chl. a}} + {\text{Chl. b}} = {\text{38.91}}{\text{A}}_{645} - {\text{14.12}}{\text{A}}_{663} (\text{mg}/{\text{g)}}$$

7 $${\text{Car }} = \left( 1000{\text{A}}_{470} - {\text{2.05Ca}} - {\text{114.8Cb}} \right){\mkern 1mu} /{\text{248 }}({\text{mg}}/{\text{g}})$$

where Total Chl. Content is total chlorophyll content, Chl. a is chlorophyll a content, Chl. b is chlorophyll b content, Car is carotenoid content, A_663_, A_645_ and A_470_ are the absorbance of chlorophyll extracts at 663 nm, 646 nm and 470 nm, respectively.

### The indices of allelopathic effects and synthetical allelopathic index

The response index (RI), referring to the method of Bruce Williamson et al.^38^:

8 $${\text{RI}} = {\text{1}} - \frac{{\text{C}}}{{\text{T}}}{\mkern 1mu} \,({\text{T}} = {\text{C}}){\text{; RI}} = \frac{{\text{T}}}{{\text{C}}} - {\text{1 (T}} < {\text{C)}}$$

where C is the control value and T is the value of different concentrations of MP and ML of *A.* *philoxeroides*; when RI < 0, it means inhibition, and the larger the absolute value, the higher the inhibition; when RI > 0, it means promotion, and the larger the absolute value, the better the promotion; the absolute value represents the strength of the allelopathy of various concentrations of MP and ML.

The synthetical allelopathic index (SE)^39^ is the arithmetic mean of the response index under different concentrations of MP and ML treatments:

9 $$\text{SE = }\frac{\left(\begin{array}{c}{\text{RI}} \, {\text{stem}} \, {\text{length}} \, \text{+} \, {\text{RI}} \, {\text{root}} \, {\text{length}} \, \text{+} \, {\text{RI}} \, {\text{nodes}} \, {\text{number}} \, \text{+} \, {\text{RI}} \, {\text{leaf}} \, {\text{number}} \, \text{+} \, {\text{RI}} \, {\text{leaf}} \, {\text{area}} \, \text{+} \, {\text{RI}}{\text{ shoot dry weight} \, } \, \, \\ \text{+} \, {\text{RI}}{\text{ root dry weight} \, } \, \text{+} \, {\text{RI}} \, {\text{ root}}-{\text{shoot}} \, {\text{ratio}} \, \text{+} \, {\text{RI}} \, {\text{plant}} \, {\text{water}} \, {\text{content}} \, \text{+} \, {\text{RI}} \, {\text{degree}} \, {\text{of}} \, {\text{succulence}}\end{array}\right)}{10}$$

when SE < 0, it means that different concentrations of MP and ML have an inhibitory effect on *A.* *philoxeroides*; when SE > 0, it means that it has a positive effect.

### Plant stable isotope ^13^C determination

The roots, stems and leaves of ^13^C-labeled 1 mmol/L MP treated *A.* *philoxeroides* were dried, ground in a mortar and pestle and passed through a 50 mesh sieve. 0.5 g of the sample was weighed and wrapped in dry tinfoil, and then the δ^13^C (‰) value of the sample was determined using an Isotope Ratio Mass Spectrometer (Thermo Fisher Scientific, model MAT-253, Germany). The following formula was used to calculate the correlation between the proportion of carbon storage, atomic percentage excess of ^13^C and biomass ratio and ^13^C content:

10 $${\text{C}} _\text{ stock} = \frac{{{\text{C}} \times {\text{M}}}}{{10^4}}$$

where C represents the percentage of carbon in different parts of plants (%); M represents the biomass of different parts of the plant^40^:

11 $${\text{R}}_\text{ sample} = \left( {\frac{{{{\delta}}^{13}{\text{C}}}}{{1000}} \times {\text{R}}_\text{PDB}} \right) + {\text{R}_\text{PDB}}$$

where R_PDB_ = 0.011237, PDB standard: PDB(Pee Dee Belemnite) is a Cretaceous marine fossil, *Belemnitella* *americana*, collected from the Pee Dee formation in South Carolina, which carbon and oxygen isotopic compositions of carbonate rocks are usually determined using PDB standards^41^:

12 $${}^{{13}}{\text{C}}_{{{\text{atom}}}} \% = \frac{{{\text{R}}_{{{\text{sample}}}} }}{{{\text{R}}_{{{\text{sample + 1}}}} }}{{ \times 100}}$$

where R_sample_ is the isotope ratio of the sample

13 $${\text{APE = }}{}^{{13}}{\text{C}}_{{{\text{atom}}}} \% \,({\text{labeled sample}}) - {}^{{13}}{\text{C}}_{{{\text{atom}}}} \% \,({\text{control sample}})$$

where ^13^C_atom_% (labeled sample) represents the atomic percentage of ^13^C measured in the labeled sample; ^13^C_atom_% (control sample) represents the atomic percentage of ^13^C measured in the unlabeled sample^42^:

14 $$^{13}{\text{C}_\text{ content}}\text{ = C }_{\text{stock}} \times \text{APE} \times 10^{3}$$

where C_stock_ represents the C amount in different parts of plants, APE represents the atom percent excess of ^13^C in different parts of plants exceeds^42^.

### Statistical analysis

The raw data were organized and statistically analyzed using Excel 2016, and one-way ANOVA was performed on the experimental treatment data using SPSS26. The effects of different concentrations of MP and ML on the response variables (Morphological indicators, biomass, physiology and ^13^C-related indicators of different parts of the plant) of *A.* *philoxeroides* were assessed using LSD and Duncan's method. The relationship between ^13^C content and biomass ratio of root, stem, leaf and total biomass was analyzed using one-dimensional linear regression. Origin2022 was used for plots and analyses to determine the extent of the effects of MP and ML on different variable indicators of *A.* *philoxeroides*.

## Results

### Effect of allelochemicals on the morphological characteristics

Different concentrations of MP had significant effects on stem length (F = 10.63, *P* < 0.001) and leaf area (F = 12.88, *P* < 0.001) of *A.* *philoxeroides*, and the most significant inhibition of leaf area was observed with MP1 (77.72%) (Table 1). with a higher concentration of MP solution (0.1–1 mmol/L), root length was shorter with high concentration inhibition, and MP1 had a significant inhibition of root length (35.58%) compared to the control. Except for MP0.1 concentration, the node number and leaf number decreased with increasing concentration. Different concentrations of ML had significant effects on stem length and leaf area, and ML1 treatment had the greatest inhibition of leaf area (73.87%). Root length, node number and leaf number were inhibited by ML (0.1–1 mmol/L) compared to the control, and the inhibition tended to diminish and then increase. ML1 had the strongest inhibitory effect on root length and leaf number (48.33% and 38.68%), and ML0.1 had the most significant inhibition on node number (21.67%).

**Table 1:** Effect of different concentrations of MP and ML on morphological indexes of *A.* *philoxeroides.*

Treatment	Stem length (cm)	Root length (cm)	Node number (ind)	Leaf number (pcs)	Leaf area (cm^2^)
CK	19.34 ± 0.61a	6.12 ± 0.43a	36.00 ± 0.63ab	69.80 ± 3.15a	6636.86 ± 263.61a
MP0.01	13.97 ± 1.00b	5.48 ± 0.55ab	34.80 ± 1.77abc	59.40 ± 2.20ab	3617.58 ± 292.76bc
MP0.1	14.74 ± 0.59b	5.69 ± 0.52ab	38.80 ± 1.96a	67.00 ± 6.89a	3782.68 ± 249.75b
MP0.5	11.89 ± 0.52bc	5.68 ± 0.33ab	32.40 ± 2.11bcd	52.80 ± 4.12bc	2580.18 ± 315.18bcde
MP1	12.41 ± 0.70bc	3.94 ± 0.45c	28.00 ± 2.76d	45.40 ± 3.33c	1478.48 ± 378.85e
ML0.01	14.37 ± 0.77b	5.28 ± 0.39ab	34.00 ± 1.41abc	62.80 ± 3.40ab	3429.94 ± 381.41bcd
ML0.1	8.50 ± 1.15d	3.87 ± 0.25c	27.40 ± 1.50d	51.00 ± 4.09bc	2273.66 ± 333.65de
ML0.5	11.96 ± 1.23bc	4.36 ± 0.34bc	30.20 ± 1.98 cd	53.80 ± 5.02bc	2663.70 ± 697.14bcde
ML1	10.63 ± 1.41 cd	3.16 ± 0.50c	28.20 ± 1.02d	42.80 ± 3.84c	1734.22 ± 491.13cde

MP0.01, MP0.1, MP0.5, MP1, representing 0.01, 0.1, 0.5, 1 mmol/L of methyl palmitate, ML0.01, ML0.1, 0.5 ML, ML1 representing 0.01, 0.1, 0.5, 1 mmol/L of methyl linolenate; data in the table are the mean ± SE, and different lowercase letters in same column indicate significant differences (*p* < 0.05).

### Effect of allelochemicals on the biomass

Different concentrations of MP and ML significantly affected the shoot dry weight on *A.* *philoxeroides* compared to the control (Table 2). The inhibition increased with increasing concentration, and MP1 and ML1 were extremely inhibitory (50.91% and 50.91%). Different concentrations of MP and ML had an inhibitory effect on root dry weight, and the most inhibitory effect of MP0.5 and ML1 was significant (40.74% and 33.33%). Compared to control, root–shoot ratio, plant water content and degree of succulence were promoted, and ML significantly increased plant water content and degree of succulence.

**Table 2:** Effects of different concentrations of MP and ML on the biomass indexes of *A.* *philoxeroides*.

Treatment	Shoot dry weight(g)	Root dry weight(g)	Root–shoot ratio	Plant water content	Degree of succulence
CK	0.55 ± 0.07a	0.27 ± 0.02 ab	0.50 ± 0.03c	0.70 ± 0.01d	3.32 ± 0.14d
MP0.01	0.42 ± 0.04b	0.22 ± 0.04bc	0.52 ± 0.04bc	0.74 ± 0.01bcd	3.90 ± 0.16abcd
MP0.1	0.42 ± 0.02b	0.30 ± 0.02a	0.70 ± 0.01ab	0.73 ± 0.01bc	3.80 ± 0.19bcd
MP0.5	0.29 ± 0.02c	0.16 ± 0.02c	0.55 ± 0.06abc	0.75 ± 0.01abc	4.05 ± 0.22abc
MP1	0.27 ± 0.04c	0.17 ± 0.02c	0.64 ± 0.08abc	0.70 ± 0.03 cd	3.50 ± 0.30 cd
ML0.01	0.42 ± 0.02b	0.22 ± 0.04bc	0.52 ± 0.07bc	0.79 ± 0.02a	4.31 ± 0.28ab
ML0.1	0.30 ± 0.02bc	0.21 ± 0.02bc	0.70 ± 0.04ab	0.77 ± 0.00ab	4.27 ± 0.05ab
ML0.5	0.30 ± 0.05bc	0.19 ± 0.01c	0.65 ± 0.06abc	0.77 ± 0.01ab	4.46 ± 0.15a
ML1	0.27 ± 0.04c	0.18 ± 0.01c	0.73 ± 0.09a	0.77 ± 0.01ab	4.31 ± 0.15ab

MP0.01, MP0.1, MP0.5, MP1, representing 0.01, 0.1, 0.5, 1 mmol/L of methyl palmitate, ML0.01, ML0.1, 0.5 ML, ML1 representing 0.01, 0.1, 0.5, 1 mmol/L of methyl linolenate; data in the table are the mean ± SE, and different lowercase letters in same column indicate significant differences (*p* < 0.05).

### Effect on photosynthetic pigment content of leaves

**Figure 1:**
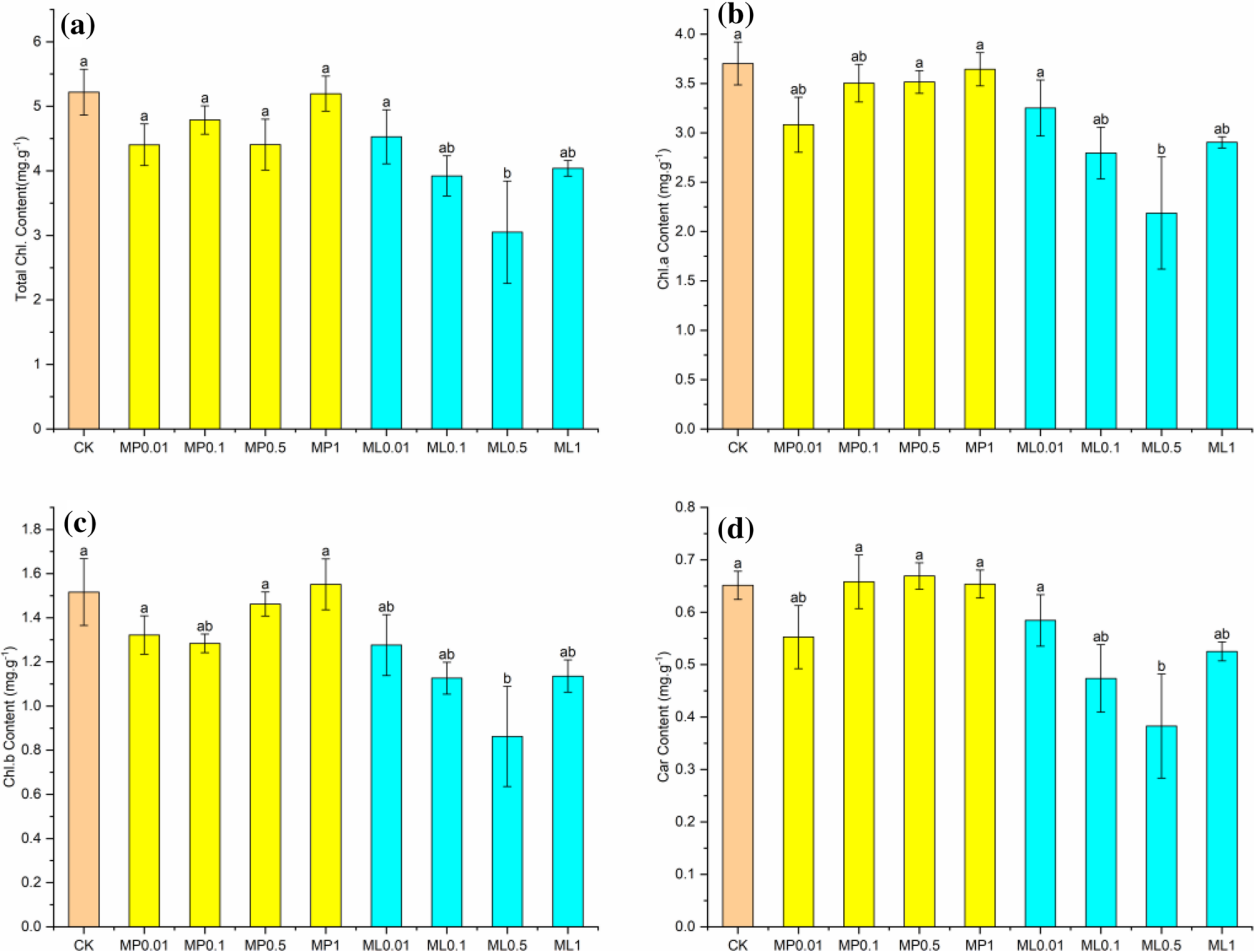
Effect of different concentrations of MP and ML treatment on total chlorophyll (**a**), chlorophyll a (**b**), b (**c**) and carotenoids (**d**) in leaves of *A.* *philoxeroides.* MP0.01, MP0.1, MP0.5, MP1, representing 0.01, 0.1, 0.5, 1 mmol/L of methyl palmitate, ML0.01, ML0.1, 0.5 ML, ML1 representing 0.01, 0.1, 0.5, 1 mmol/L of methyl linolenate; Total Chl. Content is total chlorophyll content, Chl. a is chlorophyll a content, Chl. b is chlorophyll b content, Car is carotenoid content. Data in the graphs are mean ± SE, and different lowercase letters in same column indicate significant differences (p < 0.05).

Apart from ML0.5 concentration, there was no significant effect of MP and ML on leaf of total chlorophyll, chlorophyll a, b and carotenoid contents of *A.* *philoxeroides* compared with the control (Fig. 1). The total chlorophyll content showed a trend of increasing, then decreasing and then increasing with the increase of MP concentration (Fig. 1a). As the concentration of ML increased the total chlorophyll content decreased and then increased, and the ML0.5 treatment had the lowest content (41.56%). All were smaller than the control content. Chlorophyll a content increased with rising MP concentration (Fig. 1b), and chlorophyll b and carotenoid contents of leaves treated with different MP concentrations did not differ significantly (Fig. 1c,d). Leaf chlorophyll a and b content declined with higher concentration of ML. ML0.5 significantly inhibited total chlorophyll content, chlorophyll a, b and carotenoids of *A.* *philoxeroides* (41.55%, 40.92%, 43.11% and 26.85%).

### Effect of allelochemicals on MDA levels and the enzymatic activity of leaves

Different concentrations of MP and ML had no significant effect on MDA content of *A.* *philoxeroides* leaves, and different concentrations of ML had non-significant effect on CAT and POD enzyme activities of leaves (Table 3). Treatment with different concentrations of MP significantly promoted leaf CAT enzyme activity, with MP0.01 increasing 254.12% compared to the control. Compared to CK, there was a meaningful increase in leaf POD activity by MP0.01 and MP0.1 with 102.00% and 129.59%, and the promotion effect diminished with increasing MP concentration. SOD activity of *A.* *philoxeroides* leaves was significantly inhibited by MP0.01, MP0.1 and ML0. 1 concentrations with 69.44%, 88.68% and 74.32% inhibition, respectively.

**Table 3:** Effects of different concentrations of MP and ML treatments on the enzymatic activity of the leaves of *A.* *philoxeroide.*

Treatment	MDA	CAT	POD	SOD
CK	0.27 ± 0.03abc	0.78 ± 0.27d	3.70 ± 0.24cde	11.02 ± 1.65ab
MP0.01	0.39 ± 0.06a	2.76 ± 0.18a	8.95 ± 0.49a	3.37 ± 0.28cde
MP0.1	0.35 ± 0.05ab	2.33 ± 0.32ab	7.32 ± 0.56ab	1.25 ± 1.65e
MP0.5	0.28 ± 0.01abc	2.28 ± 0.33ab	5.87 ± 1.26bc	12.22 ± 2.96ab
MP1	0.22 ± 0.02c	1.82 ± 0.30abc	4.74 ± 0.58bcd	13.51 ± 1.58a
ML0.01	0.23 ± 0.04bc	1.36 ± 0.53bcd	2.36 ± 1.32de	7.96 ± 1.15bcd
ML0.1	0.30 ± 0.05abc	0.86 ± 0.27 cd	1.35 ± 1.09e	2.83 ± 0.65de
ML0.5	0.23 ± 0.01bc	1.34 ± 0.30bcd	1.32 ± 1.13e	7.04 ± 0.94bcd
ML1	0.29 ± 0.03abc	1.76 ± 0.29abcd	5.10 ± 1.20bcd	8.68 ± 2.80abc

MP0.01, MP0.1, MP0.5, MP1, representing 0.01, 0.1, 0.5, 1 mmol/L of methyl palmitate, ML0.01, ML0.1, 0.5 ML, ML1 representing 0.01, 0.1, 0.5, 1 mmol/L of methyl linolenate; data in the table are the mean ± SE, and different lowercase letters in same column indicate significant differences (*p* < 0.05).

### The indices of allelopathic effects at different levels on *A. philoxeroides*

The response index (RI) was used to evaluate the overall allelopathic effect of MP and ML on *A.* *philoxeroides* by the indicators of stem length, root length, node number, leaf number, leaf area, shoot dry weight, root dry weight, root–shoot ratio, plant water content and degree of succulence (Table 4). As shown by the RI means, stem length, root length, node number, leaf number, leaf area, shoot dry weight, root dry weight, had negative values (− 0.13 to − 0.59) and root–shoot ratio, plant water content and degree of succulence had positive values (0.07–0.18). The synthetical allelopathic index (SE) of different concentrations of MP and ML were negative (− 0.04 to − 0.26), indicating that different concentrations of MP and ML had inhibitory effects on the growth of *A.* *philoxeroides*. MP1 and ML1 had the smallest SE (− 0.26 and − 0.24, respectively) and the strongest inhibition. The SE (0.1–1 mmol/L) reduced with the increase of MP concentration. It tended to decline and then increase and then decrease with the increase of ML concentration.

**Table 4:** Response index of different concentrations of MP and ML treatments on *A.* *philoxeroides.*

Treatment	Stem length	Root length	Node number	Leaf number	Leaf area	Shoot dry weight	Root dry weight	Root–shoot ratio	Plant water content	Degree of succulence	SE
MP0.01	− 0.28 ± 0.05a	− 0.11 ± 0.05ab	− 0.04 ± 0.06ab	− 0.15 ± 0.03ab	− 0.45 ± 0.05ab	− 0.22 ± 0.07a	− 0.19 ± 0.07b	0.03 ± 0.1a	0.06 ± 0.02ab	0.14 ± 0.06ab	− 0.12
MP0.1	− 0.24 ± 0.04a	− 0.07 ± 0.1ab	0.02 ± 0.08a	− 0.04 ± 0.11a	− 0.43 ± 0.03a	− 0.2 ± 0.06a	0.09 ± 0.05a	0.28 ± 0.05a	0.05 ± 0.03ab	0.11 ± 0.07ab	− 0.04
MP0.5	− 0.38 ± 0.03ab	− 0.05 ± 0.1a	− 0.1 ± 0.06abc	− 0.23 ± 0.08ab	− 0.61 ± 0.06abc	− 0.45 ± 0.07a	− 0.42 ± 0.04b	0.06 ± 0.11a	0.07 ± 0.03ab	0.17 ± 0.06ab	− 0.19
MP1	− 0.36 ± 0.02ab	− 0.34 ± 0.1bc	− 0.22 ± 0.08bc	− 0.34 ± 0.07b	− 0.78 ± 0.06c	− 0.46 ± 0.1a	− 0.32 ± 0.13b	0.18 ± 0.08a	0.01 ± 0.03b	0.04 ± 0.06b	− 0.26
ML0.01	− 0.25 ± 0.05a	− 0.13 ± 0.03ab	− 0.05 ± 0.05abc	− 0.09 ± 0.08a	− 0.47 ± 0.08ab	− 0.19 ± 0.08a	− 0.19 ± 0.12b	0 ± 0.12a	0.12 ± 0.03a	0.31 ± 0.08a	− 0.10
ML0.1	− 0.56 ± 0.07c	− 0.35 ± 0.07bc	− 0.24 ± 0.04c	− 0.27 ± 0.06ab	− 0.65 ± 0.06bc	− 0.43 ± 0.05a	− 0.21 ± 0.02b	0.28 ± 0.06a	0.09 ± 0.02ab	0.22 ± 0.03ab	− 0.21
ML0.5	− 0.38 ± 0.07ab	0.27 ± 0.08abc	0.16 ± 0.06abc	− 0.22 ± 0.09ab	− 0.58 ± 0.12abc	− 0.41 ± 0.13a	− 0.29 ± 0.07b	0.19 ± 0.1a	0.1 ± 0.02a	0.25 ± 0.04a	− 0.18
ML1	− 0.45 ± 0.06bc	− 0.45 ± 0.12c	− 0.21 ± 0.04bc	− 0.38 ± 0.07b	− 0.74 ± 0.07abc	− 0.44 ± 0.16a	− 0.31 ± 0.08b	0.25 ± 0.13a	0.09 ± 0.02ab	0.22 ± 0.05ab	− 0.24
Mean	− 0.36	− 0.22	− 0.13	− 0.22	− 0.59	− 0.35	− 0.23	0.16	0.07	0.18	

MP0.01, MP0.1, MP0.5, MP1, representing 0.01, 0.1, 0.5, 1 mmol/L of methyl palmitate,ML0.01, ML0.1, 0.5 ML, ML1 representing 0.01, 0.1, 0.5, 1 mmol/L of methyl linolenate; data in the table are the mean ± SE, and different lowercase letters in same column indicate significant differences (*p* < 0.05).

### Carbon storage in different parts of *A. philoxeroides*

**Figure 2:**
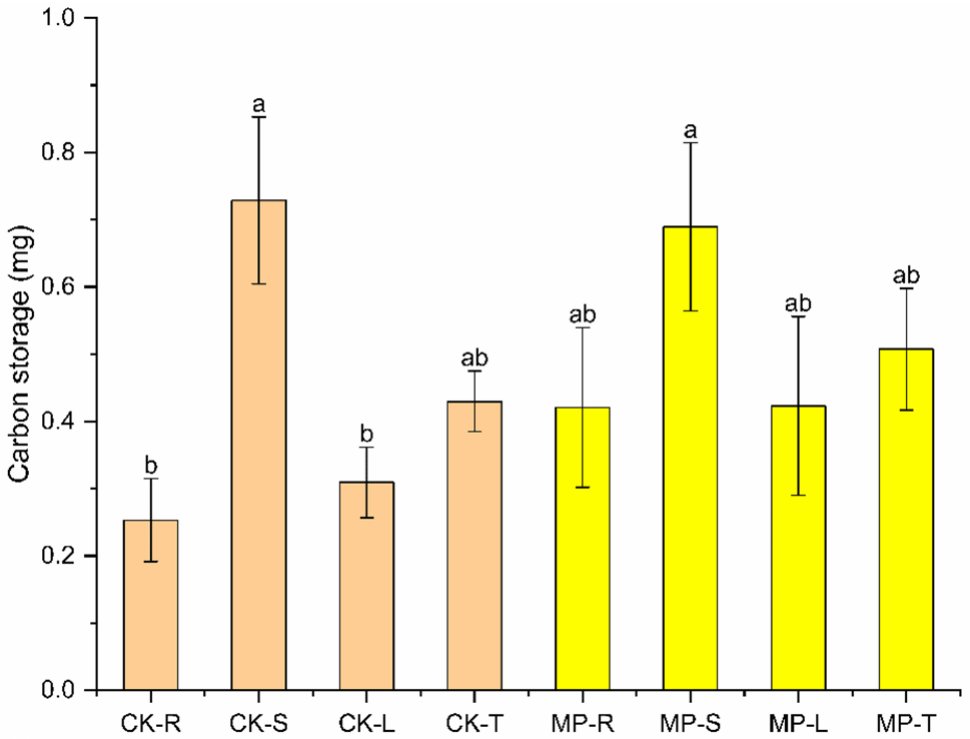
Effects of different treatments on carbon storage in roots, stems, leaves and total plant of *A.* *philoxeroides.* The CK-R, CK-S, CK-L, and CK-T represent the root, stem, leaf, and total plant of the control group; The MP-R, MP-S, MP-L, and MP-T represent the root, stem, leaf, and total plant of 1 mmol/L MP treatment.

Carbon stocks of CK treatments of *A.* *philoxeroides* stems were significant (p < 0.05) compared to roots, leaves and total plant, and carbon stocks of CK and MP roots, stems, leaves and total plant showed the same trend (Fig. 2). The CK group and the MP group treated stems had the largest carbon stocks, the carbon stocks of roots and leaves were equal, the carbon stocks of stems were about twice as large as those of roots and leaves, and the carbon stocks of the total plant were larger in the MP treatment than in the CK. The carbon stocks of different parts of MP treated plants were insignificant, but the carbon stocks of stems in CK treatment were significantly greater than those of roots and leaves, and increased by 65.22% and 57.52%, respectively.

### Atom percent excess of different parts of *A. philoxeroides*

**Figure 3:**
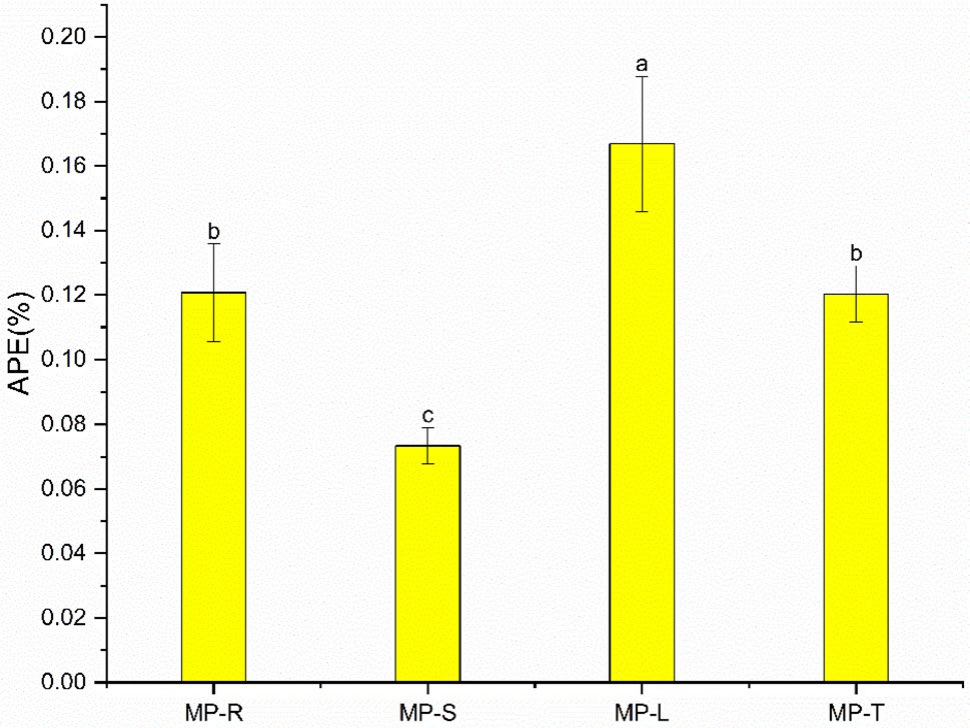
Effect of MP treatment on root, stem, leaf and total plant APE of *A.* *philoxeroides.* The MP-R, MP-S, MP-L, and MP-T represent the root, stem, leaf, and total plant of 1 mmol/L MP treatment groups; The APE is the atom percent excess (%).

With MP treatment, there was a highly significant effect (p < 0.05) on the atom percent excess (APE) between roots, stems and leaves of *A.* *philoxeroides* (Fig. 3). Compared to the total plant, both stems and leaves were significantly affected, excluding the APE of roots which was not significant. The average APE size of the roots, stems and leaves was in the order of leaves (0.17%) > roots (0.12%) > stems (0.07%). APE was significantly lower by 27.64% and 56.01% for roots and stems, respectively, compared to leaves, and by 39.21% for stems compared to roots. Compared to total plant APE, leaves significantly increased by 38.65% and stems significantly decreased by 39.01%.

### Correlation analysis of root, stem, leaf biomass ratio and total biomass with ^13^C content

**Figure 4:**
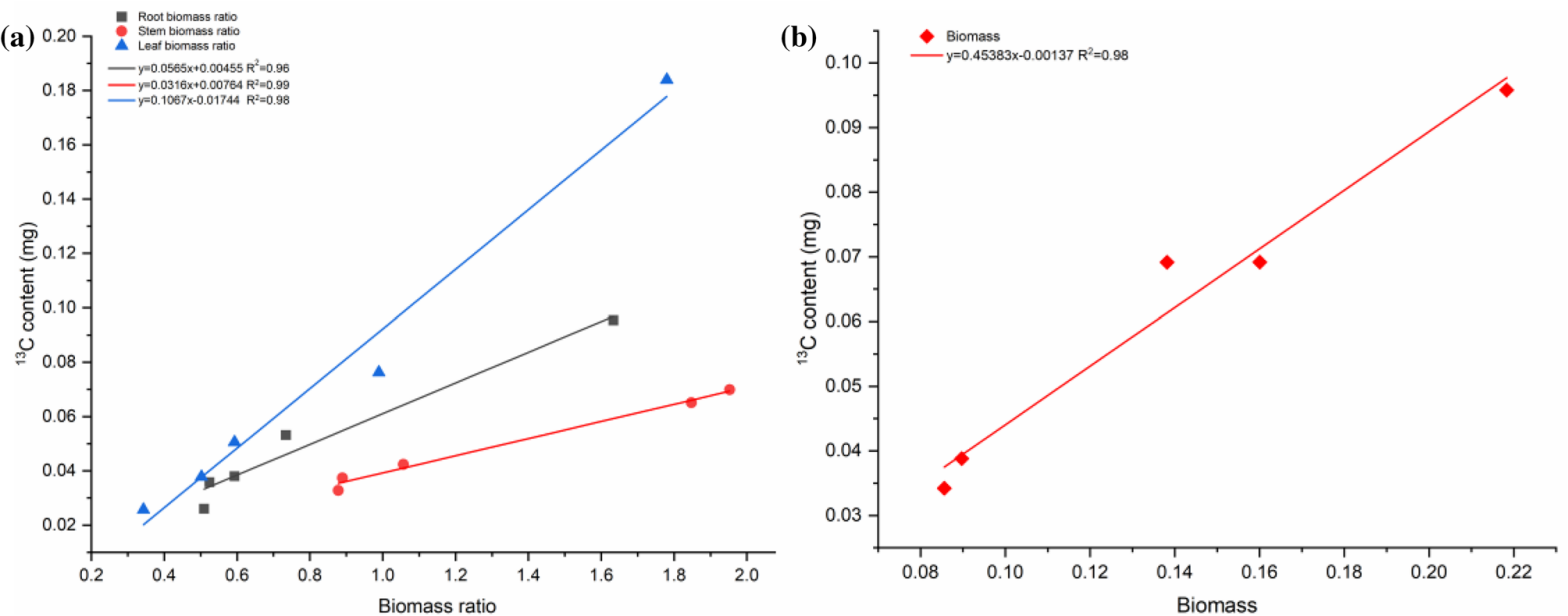
Correlation fit of root, stem and leaf biomass ratio and total biomass with ^13^C content in *A.* *philoxeroides*. Biomass ratios: root biomass (solid squares; solid lines); stem biomass (solid circles; double dotted lines); leaf biomass (solid triangles: dotted lines); R^2^ correlation factor. Biomass: total plant biomass (solid squares; solid lines); R^2^ correlation factor.

A linear regression of the root, stem and leaf biomass ratios of *A.* *philoxeroides* on the associated ^13^C content showed a highly significant positive correlation (*p* < 0.01) (Fig. 4). The R^2^ of root, stem, leaf and total biomass fitted by the model were all above 0.95, where the linear coefficients were ranked in order of magnitude as R^2^ stem (0.99) > R^2^ leaf (0.98) = R^2^ total (0.98) > R^2^ root (0.96). The average increase was ranked in order of total plant (0.4538) > leaf (0.1067) > root (0.0565) > stem (0.0316). Overall, the one-dimensional linear regression of root, stem and leaf biomass ratios, total biomass and ^13^C content were fitted with high accuracy, and the ^13^C content tended to increase with increasing biomass, which could predict the ^13^C content of *A.* *philoxeroides*.

## Discussion

*Alternanthera* *philoxeroides* is a malignant weed with strong invasive properties, and the indigenous plants are able to secrete secondary metabolites into the surrounding area, producing allelopathic inhibition, whose effects are mainly reflected in seed germination and seedling growth^14^. Therefore, we selected methyl palmitate (MP) and methyl Linolenate (ML), the most representative fatty acid methyl esters of *Humulus* *scandens* root extracts^43–45^, as exogenous allelopathic substances to investigate their effects on the growth morphology, biomass and physiological indexes of seedlings of *A.* *philoxeroides*. ^13^C labeled the strongest allelopathy of 1 mmol/L MP (SE = − 0.26), revealing the relationship between the distribution of allelopathic substances in the roots, stems and leaves of invasive weeds and the strength of the allelopathic effect, so as to provide a new means for the control of invasive weeds. The study found that the exogenous allelochemicals MP and ML had certain allelopathic inhibitory effects on the stem length, root length, node number, leaf number and leaf area of seedlings of *A.* *philoxeroides* compared with the control. The principal reason is that the allelochemicals can prevent roots from absorbing soil nutrients, reduce the connection between stem nodes, roots and leaves, affect photosynthesis of leaves and reduce photosynthetic efficiency^46–49^. Inhibition of root length, node number and leaf number enhanced with increasing concentration for 0.1–1 mmol/L MP, showing inhibition at high concentrations. The biomass of *A.* *philoxeroides* reflects the accumulation of plant organic matter, which is the most direct indicator of plant growth, and the organic material distribution mode of growth biomass is related to the tolerance of stressful environment^50^. The inhibition effect of shoot dry weight and root dry weight of *A.* *philoxeroides* were enhanced with increasing MP and ML concentrations, which were positively correlated with the concentration of allelochemicals. Plant roots are affected by allelochemicals, which inhibit the division and elongation of root tip cells, thus reducing the ability to absorb surrounding nutrients and affecting aboveground biomass accumulation. Another cause is that allelopathic substances are able to reduce the net primary productivity of *A.* *philoxeroides*, resulting in a reduction in belowground and aboveground biomass accumulation^51^.

Chlorophyll a and chlorophyll b absorb red light and blue-violet light respectively, and carotenoids have the function of absorbing light energy and dissipating the remaining photosynthetic energy, which are closely related to plant organic matter accumulation and growing development^52^. It was concluded that MP and ML inhibited the total chlorophyll content, chlorophyll a, b and carotenoids of *A.* *philoxeroides*, and ML0.5 inhibited the total chlorophyll most significantly. ​Presumably due to the occurrence of allelopathic stress persecution in plants, the ultrastructure of chloroplasts is harmed, leading to chlorophyll degradation, chlorophyll content reductions, light and dark reactions are affected, resulting in metabolic dysregulation and lowered photosynthetic rate^53,54^. The reduction in chlorophyll accumulation was reported to be associated with the deletion of Phosphatidylglycerol (PG), which could be a result of allelochemicals inhibiting the up-regulation of chlorophyll biosynthesis pathway genes and accelerating the induction of down-regulation of degradation pathway genes expression, resulting in less chlorophyll accumulation^55^.

Reactive oxygen species (ROS) are generated in plants under unfavorable conditions, which cause membrane lipid peroxidation to produce malondialdehyde (MDA) and damage cell membranes and macromolecules such as proteins, hence affecting normal growth and development of plants^56^. At the same time, in plants under stressful environments, there is an enzymatic protection system, namely the antioxidant defense system. Antioxidant enzymes in plants majorly include superoxide dismutase (SOD), catalase (CAT) and peroxidase (POD), which can remove the excess free radicals and maintain the balance of reactive oxygen species production and removal in plant cells^57^. Therefore, SOD, CAT, POD activity and MDA content are often used as important indicators to determine whether or not a plant is damaged by adversity and the extent of damage^58^. The research results revealed that the leaf MDA content was greater than the control under MP and ML allelopathic stress, implying that the plant cells were attacked by free radicals and the cells were harmed so that the balance of reactive oxygen species was disrupted. The activities of CAT and POD in leaves of *A.* *philoxeroides* treated with different concentrations of MP were larger than those of the control group due to the differences in exogenous allelopathic substances and solution concentrations. SOD enzyme activities were more than the control group, except for MP0.01 and MP0.1 treatments, which were smaller than the control group. The CAT and POD enzyme activities of *A.* *philoxeroides* leaves under ML concentration treatment were greater than the control, but the SOD enzyme activity was less than the control, showing that the antioxidant defense systems activity was affected by the stress concentration in addition to the exogenous allelochemicals. Different concentrations of different exogenous allelopathic substances enhanced the resistance of *A.* *philoxeroides* to a certain extent, and the increase of enzyme activity strengthened the scavenging ability against free radicals^59^. This is explained by the fact that the different concentrations of MP and ML will stimulate the leaves of *A.* *philoxeroides* to strengthen the metabolism and the activity of the antioxidant enzyme protection system in the organism under the condition of adversity. The SOD, CAT, and POD activities will be promoted to scavenge the excessive oxygen radicals in the body in order to withstand the damage of the external environment. That was the adaptive mechanism of *A.* *philoxeroides* under allelopathic stress, demonstrating that allelochemicals have a stressful effect to some extent^60^. The allelopathic substances of linoleic acid, palmitic acid and ethyl palmitate in sweet potato aqueous extracts increased CAT and POD enzyme activities and decreased SOD enzyme activities in *A.* *philoxeroides*, which is consistent with the above findings^61^.

The response index (RI) and synthetical allelopathic index (SE) represent the intensity of allelopathic effects of various plants^38,62^. In terms of the mean values of different morphological characteristics and biomass indicators, RI of all indicators were negative, apart from the positive values of plant water content, root–shoot ratio and degree of succulence RI. Leaf area > Stem length > Shoot dry weight > Root dry weight > Root length = Leaf number > Node number, where the leaf area had the slightest RI and the strongest allelopathic inhibition. As shown by the synthetical allelopathic index, the SE of MP and ML treated with different concentrations were negative, indicating that both allelopathic substances had inhibitory effects on the growth of *A.* *philoxeroides*. The magnitude of inhibition was, MP1 > ML1 > ML0.1 > MP0.5 > ML0.5 > MP0.01 > ML0.01 > MP0.1, and the MP1 (SE = − 0.26) concentration had the strongest inhibitory effect on *A.* *philoxeroides*.

The ^13^C labeling was based on the selection of 1 mmol/L MP with obvious inhibitory effect, and the distribution of ^13^C was detected in the roots, stems and leaves of *A.* *philoxeroides*, which could indicate that the exogenous allelopathic substances could be directly absorbed. In the natural state, some indigenous plants produce secondary metabolites that interact with surrounding soil microorganisms and alter the community structure of plant inter-rooted microorganisms, leading to soil colony dysbiosis and soil acidification, thus indirectly affecting the growth of invasive weeds^63,64^. But in most cases, the allelopathic substances secreted by indigenous plants are mainly absorbed and utilized by the roots of invasive weeds and participate in cellular metabolic processes to inhibit their growth and development^65^. The distribution of the atom percent excess (APE) in different parts of *A.* *philoxeroides* was different, leaf > root > stem, and plant roots and stems produced more lignin and cellulose, which reduced the synthesis and utilization of allelopathic substances; it also related to the transport of the phloem of the stem, and *A.* *philoxeroides* enhanced photosynthesis in order to resist the stress damage of allelopathic substances, which led to the accumulation of more ^13^C photosynthetic products in the leaf part^66^. The distribution of ^13^C in the different parts of *A.* *philoxeroides* showed that the distribution of allelopathic substances absorbed by the plant in roots, stems and leaves was linked to the allelopathic inhibition of morphological characteristics (stem length, root length, node number, leaf number and leaf area). The highest ^13^C content in leaves had the strongest inhibitory effect on invasive weeds. The maximum accumulation of ^13^C in leaves also indicated some inhibitory effect on pigment content and enzyme activity of leaves. Allelopathic stress affected the enzyme activity of the leaf cells of invasive weeds, causing an increase in intracellular reactive oxygen radicals (Table 3), reducing chlorophyll content on cystoid membranes (Fig. 1), and lowering light uptake and conversion. This led to impaired electron transport and oxidative phosphorylation, which reduced photosynthesis and ultimately resulted in macroscopic characteristics of delayed germination and weak seedlings^67,68^. The relationship between biomass and ^13^C content in this study showed that the biomass ratios of roots, stems and leaves of *A.* *philoxeroides* had a highly significant positive correlation (*p* < 0.01) with the ^13^C content of the corresponding parts, and the correlation coefficient between the two reached over 0.95. It means that biomass and allelopathic inhibition are closely linked, and the more biomass accumulated by plants, the more allelopathic substances are absorbed and the more obvious the inhibition is, thus reaching the critical value of biomass accumulation, which makes them grow slowly and show weakness on the plant. Gealy et al. found that the δ^13^C values of rice were negatively correlated with biomass by a linear fit, in contrast to the present study, which may be related to the differences in plants and research methods^30,68^.

## Conclusions

Exogenous allelochemicals have potential allelopathic toxic effects and have significant inhibitory effects on the morphology, biomass and physiological characteristics indexes of the growth of seedlings of *A.* *philoxeroides*. The stable isotope ^13^C-labeled methyl palmitate showed that ^13^C had different distributions in roots, stems and leaves of *A.* *philoxeroides*, specifically, leaves > roots > stems, and the root, stem and leaf biomass ratios of the invasive weed were positively related to the ^13^C content. This proves that the spread of invasive alien plants can be well controlled by developing and researching exogenous allelopathic substances from water extracts of indigenous species, which can be directly absorbed by the root system of *A.* *philoxeroides* and produce inhibitory effects. This provides research directions for the future utilization of natural herbicides. However, in the natural condition, MP and ML may be affected by abiotic factors such as climate, temperature, light, water, soil chemicophysical properties and biotic factors such as soil animals, soil microorganisms, and activities of other plants, all of which may enhance or weaken the allelopathy of exogenous allelopathic substances. Therefore, their allelopathic effects need to be further investigated under natural conditions, especially the combined effects of indigenous plants on soil physicochemical properties and inter-root microorganisms.

## Data Availability

All data used in this study can be obtained by contacting the corresponding author.
